# The Fitness Landscapes of *cis*-Acting Binding Sites in Different Promoter and Environmental Contexts

**DOI:** 10.1371/journal.pgen.1001042

**Published:** 2010-07-29

**Authors:** Ryan K. Shultzaberger, Daniel S. Malashock, Jack F. Kirsch, Michael B. Eisen

**Affiliations:** 1 Department of Molecular and Cell Biology, University of California Berkeley, Berkeley, California, United States of America; 2 Graduate Group in Comparative Biochemistry, University of California Berkeley, Berkeley, California, United States of America; 3 Department of Chemistry, University of California Berkeley, Berkeley, California, United States of America; 4 Howard Hughes Medical Institute, University of California Berkeley, Berkeley, California, United States of America; 5 California Institute of Quantitative Biosciences, University of California Berkeley, Berkeley, California, United States of America; 6 Genomics Division, Ernest Orlando Lawrence Berkeley National Laboratory, Berkeley, California, United States of America; University of Toronto, Canada

## Abstract

The biophysical nature of the interaction between a transcription factor and its target sequences *in vitro* is sufficiently well understood to allow for the effects of DNA sequence alterations on affinity to be predicted. But even in relatively simple *in vivo* systems, the complexities of promoter organization and activity have made it difficult to predict how altering specific interactions between a transcription factor and DNA will affect promoter output. To better understand this, we measured the relative fitness of nearly all *Escherichia coli* 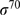 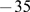 binding sites in different promoter and environmental contexts by competing four randomized 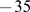 promoter libraries controlling the expression of the tetracycline resistance gene (*tet*) against each other in increasing concentrations of drug. We sequenced populations after competition to determine the relative enrichment of each −35 sequence. We observed a consistent relationship between the frequency of recovery of each −35 binding site and its predicted affinity for 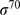 that varied depending on the sequence context of the promoter and drug concentration. Overall the relative fitness of each promoter could be predicted by a simple thermodynamic model of transcriptional regulation, in which the rate of transcriptional initiation (and hence fitness) is dependent upon the overall stability of the initiation complex, which in turn is dependent upon the energetic contributions of all sites within the complex. As implied by this model, a decrease in the free energy of association at one site could be compensated for by an increase in the binding energy at another to produce a similar output. Furthermore, these data show that a large and continuous range of transcriptional outputs can be accessed by merely changing the 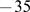, suggesting that evolved or engineered mutations at this site could allow for subtle and precise control over gene expression.

## Author Summary

A major challenge in molecular genetics has been to understand how *cis*-regulatory information is integrated to determine the amount of transcript generated. The difficulty has been that there are a large number of variables (known and unknown) that combine through an extensive array of possible mechanisms. Differences in the affinity of a binding site for its cognate binder within the initiation complex are known to account for significant differences in promoter output, but data for the activity of binding site variants *in vivo* has been limited. Here, we were able to map the fitness of nearly all *E. coli*   binding sites in multiple promoter and environmental contexts using a novel method that utilizes the sequencing power of a next generation DNA sequencer. These data for the first time show the phenotypic range and continuity of a nearly complete set of possible binding targets *in vivo*, and they are useful in our ability to understand the mechanism, evolution, and designability of gene regulation.

## Introduction

While we have a reasonable understanding of the biophysical forces that determine the affinity of a transcription factor to its target sequences [1]–[4], we still have a poor understanding of how the affinity of a factor for a site affects the output of the promoter in which it sits. The major challenge is that these relationships are highly context dependent. A high affinity site tightly bound in isolation will have no function in that it will not affect the rate of transcription of a gene, whereas a low affinity site weakly bound in the context of the initiation complex will. More subtly, a single base pair difference in the spacing between sites can affect the function of those sites [5], [6]. Here, we attempt to better understand how binding site affinity and context relate to promoter output by determining the relative fitness of 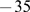 binding sites within specific variations of an engineered promoter in the bacteria *Escherichia coli*.

The engineered promoter that we use contains three binding sites: one for the transcriptional activator MarA [6], and another for the 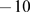 and the 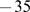 that are recognized by 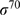 [7]. In the simplest thermodynamic model of transcriptional regulation in prokaryotes, the rate of transcriptional output varies as a direct function of the stability of the initiation complex [8]–[11]. The stability of the initiation complex in turn is dependent upon the cooperative binding of multiple DNA-binding transcription factors, each of which recognizes a degenerate set of sequences with different affinities [4]. The binding strengths of these sites are distributed such that there is a single optimal site that is bound with the highest affinity (the consensus site) and an increasing number of sequences that are bound with lower affinities as the sequences deviate from the consensus [1]–[3]. At some point the deviation becomes so great, that the site is no longer specifically bound and all remaining sequences have the same non-specific binding energy. The general assumption has been that the greater the affinity that the factor has for a site, the greater the occupancy at that site and the greater the probability that it will affect transcription [10]. This has only recently been tested for large libraries of sequences, and indeed much of the variance in expression can be explained by differences in binding site affinity [12]. Given this relationship, the distribution of binding energies for a factor defines the range of regulatory phenotypes that can be selected [2], [13], the number of possible DNA sequences that can be used to generate that phenotype, and subsequently the likelihood of a sequence of that strength evolving.

How multiple binding sites combine to determine the stability of the initiation complex is poorly understood, mainly because there are a large number of proteins that can cooperate to regulate transcription through a variety of mechanisms [9], [14], including direct stabilization or destabilization of the initiation complex through protein-protein interactions or occlusion [15], [16] or by perturbations of DNA structure that affect promoter-DNA binding [17], [18]. MarA has been shown to modulate transcription through multiple mechanisms depending on its binding context [6]. Here we use MarA as a Class I activator that increases the rate of expression by stabilizing interactions with the carboxy-terminal domain of the alpha subunit (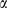
CTD) [6], [9], [19]. The ordering, spacing and orientation of binding sites can also mediate transcriptional regulation [11], [20]. Differences in the spacing between the 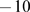 and the 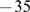 [5], [21] and between MarA and the 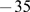 have been shown to affect the rate of transcription [6].

Here, we examine the effects of varying a binding site on promoter output by measuring the relative fitness of 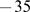 binding sites in different promoter and environmental contexts. To do this we placed the tetracycline resistance gene under control of the MarA-activated 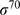 promoter on the plasmid pBR322. We generated four libraries that contained different strength 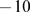 and MarA binding sites, to yield four varied energetic contexts for selection. By increasing the tetracycline concentration, we can change the range of selected viable transcriptional outputs. We competed variants within a library in liquid culture for 24 hours, and sequenced the competed population with an Illumina Solexa sequencer. Using this approach, we were able to map the fitness of a large population of binding sites in multiple promoter and environmental contexts relatively easily.

## Results

### Selection system

**Figure 1:**
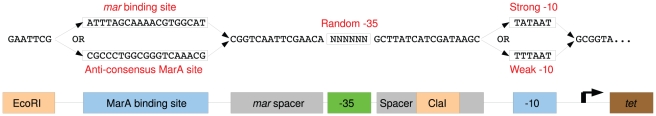
Schematic Diagram of selection promoter. Sequences of the four randomized 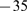 promoter libraries (top), and a diagram mapping the promoter components (bottom). The MarA or 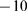 sites were varied (blue boxes). Spacing between the binding sites may affect transcriptional output [5]. We used the same sequence between the 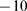 and 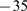 found in the *tet* promoter of pBR322 in our selection system because it has the optimal spacing [11]. We used a slight variation of the spacer between the MarA binding site and the 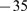 from the *mar* gene [22]. Spacer sequences are shown in gray. Restriction sites used to clone synthesized libraries into the selection plasmid are marked in orange. All libraries have 6 randomized bases at the 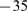 hexamer (green box).

We generated four plasmid libraries that contained the tetracycline resistance gene (*tet*) under the control of a MarA-activated 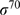 promoter with a randomized 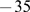 binding site. Each library contained a different combination of 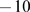 and MarA binding sites (Figure 1). The 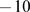 was either the consensus (TATAAT) or the weaker variant (TTTAAT). The MarA binding site was either the one that regulates the *mar* operon [22], or the anti-consensus site, which is not expected to bind or be activated by MarA. We will refer to each library based on which MarA binding site (Mar or Anti), and which 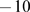 binding site (TAT or TTT) it contains. The four libraries therefore are named Mar:TAT, Anti:TAT, Mar:TTT and Anti:TTT.

To test the dependency of cell growth in tetracycline on the sequence at the 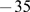, we created promoters that contained either the consensus 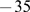 TTGACA or the anti-consensus 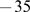 GCCGGC in the Mar:TTT context. The anti-consensus site did not allow growth at as low as 5 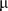
g/ml of tetracycline, where the consensus 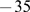 allowed for growth in tetracycline concentrations at least as high as 100 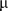
g/ml suggesting that cell survival is dependent upon the 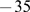 binding site (data not shown).

### 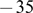 binding site competitions

Promoter competitions were performed as described in Materials and Methods. Briefly we transformed each library into *E. coli* cells and grew the cells overnight. The following morning, fresh LB cultures containing increasing concentrations of tetracycline were inoculated with the overnight cultures. Cells were competed for 24 hours and the competed populations were sequenced on a Solexa sequencer to determine the relative frequency of each 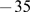 hexamer. We sequenced 24 competed populations that covered 20 distinct 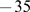 selection conditions. Each competed population is named based on the competed library and on the concentration of tetracycline used in the competition. We carried out two independent competitions with the Mar:TAT and Mar:TTT libraries. The first was performed over the range of 5 to 30 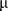
g/ml tetracycline. We expanded the range to 50 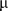
g/ml tetracycline for all other experiments. To distinguish between different competitions with the same library, each culture that came from the same starter is given a common number (1 or 2). For example, Mar:TAT Tet-5 (1) and Mar:TAT Tet-10 (1) came from the same Mar:TAT overnight culture, but Mar:TAT Tet-50 (2) came from a different one.

The number of sequencing reads are given in Table S1. Differences in read numbers are most likely a result of sample loss in the Solexa prep and to the lower cell density in higher tetracycline concentrations, especially with libraries containing the TTT 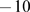. All but four of the sequenced competed populations had at least 25,000 reads. As expected, Mar:TAT Tet-5 (1) was the most variable, and appeared to show only a slight preference for the sequence at the 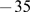 binding site. We observed 3918 of the 4096 possible 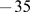 hexamers in this population, suggesting that the coverage of all 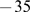 sequences in our library is essentially complete.

We sequenced Anti:TAT Tet-5 (1) and Mar:TTT Tet-5 (2) on two independent sequencing runs to determine if the number of sequenced DNA molecules gave an accurate and reproducible representation of the competed promoter populations. These runs generated 29,803 and 93,863 reads for the Anti:TAT Tet-5 (1) library and 33,229 and 11,263 reads for the Mar:TTT Tet-5 (2) library. We compared the relative frequency of each 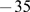 as determined from sequencing run 1 against run 2 and observed an 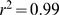 for both samples (data not shown). This suggested that for the more degenerate TAT libraries, as few 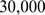 reads sufficiently covers the distribution of 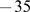 binding sites. As few as 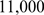 reads are sufficient for the TTT libraries.

**Figure 2:**
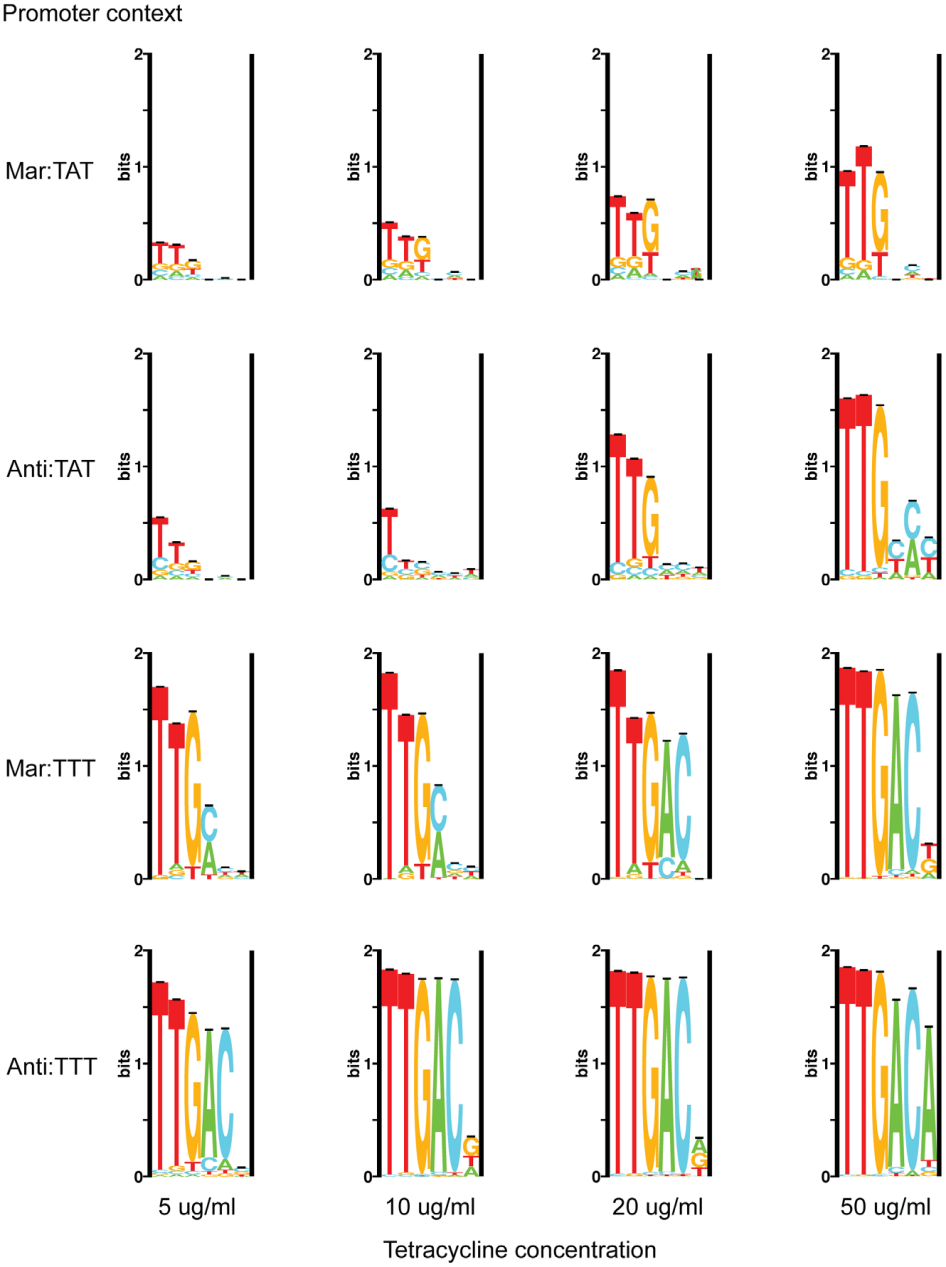
Sequence logos for competed 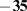 populations. Sequence logos show the amount of variability in 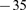 binding sites under different selective conditions [33]. The library used in each selection is reported to the left of the corresponding logos and the tetracycline concentration is given below.

Sequence logos are shown for the population of 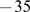 binding sites from each promoter context at 5, 10, 20 and 50 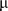
g/ml tetracycline (Figure 2). Logos generated from the Mar:TAT (1) and Mar:TTT (1) competitions over the smaller range of 5 to 30 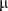
g/ml were similar (data not shown). We observed a decrease in the variability for each library as the amount of tetracycline used for selection was increased, with the population converging towards the consensus binding site TTGACA, suggesting that only stronger sites (those closer to the consensus) are viable under more stringent selection conditions. We observed a similar decrease in variability as we decreased the energetic contribution of the other components in the promoter, strongly suggesting that a decrease in the affinity of the 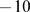 or MarA binding sites can be compensated by an increase in the strength of the 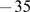. The single base-pair mutation in the 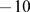 had a major effect on the population variability. Whereas completely destroying the MarA binding site by replacing it with the anti-consensus affected the population variability considerably less.

For most populations, the first position of the hexamer is the least variable, and the site increases in variability towards the 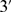 end. The first three positions are much more conserved than the last three, and position 6 appears to be relatively non-specific for most populations. This is consistent with the 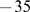 logo made from naturally occurring 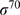 sites [11]. Only at the most stringent selective condition (Anti:TTT Tet-50) does the consensus sequence dominate.

**Figure 3:**
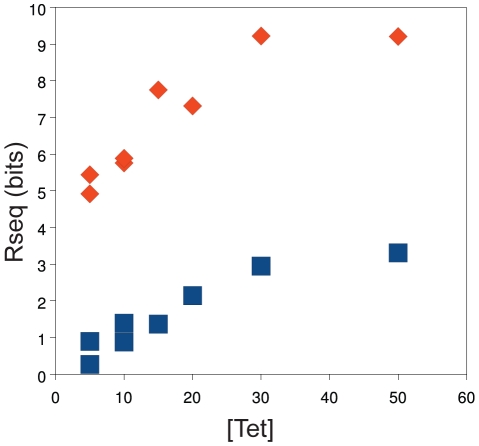
Population information content increases as a function of tetracycline concentration. The concentration of tetracycline (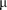
g/ml) used in the selection is on the x-axis. The information content (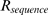) of the competed population is on the y-axis [23]. Data for the Mar:TAT (blue squares) and the Mar:TTT (red diamonds) libraries are shown.

We compared the information content (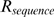) [23] for each competed population as a function of tetracycline concentration for the Mar:TAT and Mar:TTT libraries (Figure 3). This figure includes data for both competition series with these libraries. Both libraries show a linear increase in information content from 5 to 30 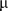
g/ml, with a leveling at 50 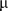
g/ml. As apparent from the sequence logos in Figure 2, the information content of the Mar:TTT library is much greater than that of the Mar:TAT library at all concentrations of tetracycline, suggesting that a weaker 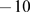 needs to be compensated for by a stronger 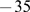 for the promoter to be viable. Duplicate selections at 5 and 10 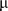
g/ml showed similar information contents for both libraries.

### 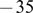 fitness as a function of binding affinity

**Figure 4:**
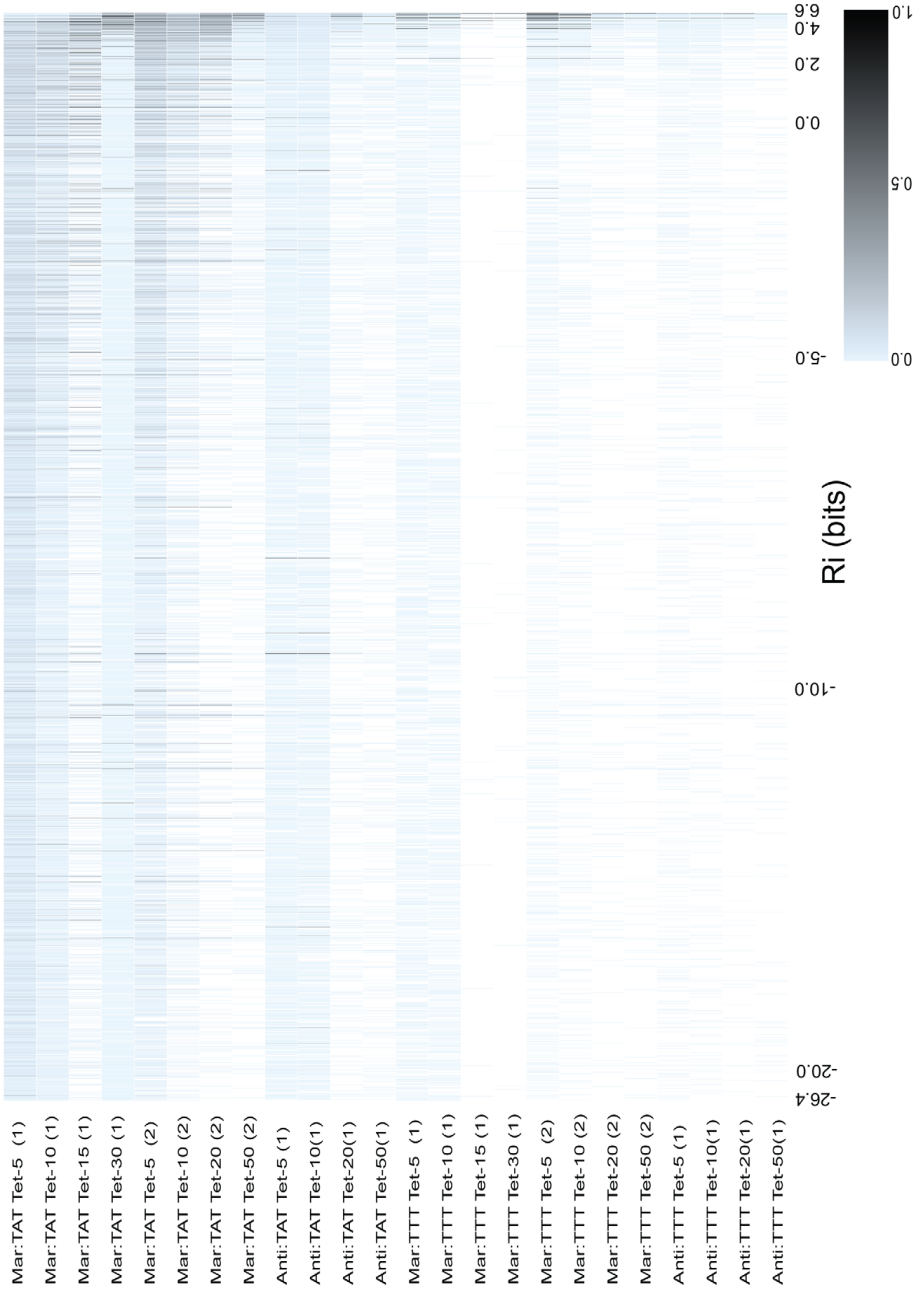
Relative fitness of all 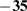 binding sites as a function of binding site strength. The 4096 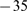 binding sites were ranked according to their predicted affinity (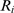) from weakest to strongest. The 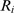 value for major intervals are written on the x-axis. 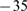 hexamers that were not observed in a competition are shown as white boxes, and all hexamers that occurred at least once are shown as blue boxes that increase in saturation to black as they increase in relative fitness. The relative fitness for a 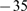 is the the number of reads containing that 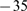 divided by the number of reads of the most frequently observed 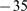 for an individual competition. A scale is given in the bottom right corner to show the saturation for a given relative fitness. Each column represents data for a different competition experiment. The name of the competed population is given to the left of each column.

We predicted the relative affinity (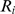) of 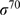 to each 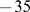 using the information theory based approach described in [2], [4] and the 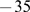 model presented in [11] (see Materials and Methods). The sites ranged in strength from 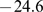 to 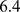 bits of information. Conventionally, sites with more than 0 bits are thought to be specifically bound [24]. 418 of the 4096 binding sites were 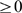 bits. The relative fitness of each 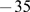 in the population was calculated by dividing the number of occurrences of that 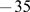 by the number of occurrences of the most frequently observed 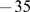. We ranked all 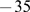 binding sites according to their 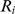, and compared the relative frequency for each 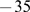 in each experiment in Figure 4, and only those sites with an 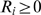 bits in Figure S1.

The majority of 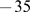 hexamers were present in all libraries that contained the 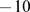 sequence TATAAT. As seen in Figure 2, there is a decrease in the variability of observed 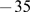 binding sites as we increased the concentration of tetracycline used in selection and as the strengths of the 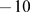 and MarA sites are decreased in the promoter. We also observed a convergence of the viable sites towards those with higher information (sites closer to the consensus sequence).

**Figure 5:**
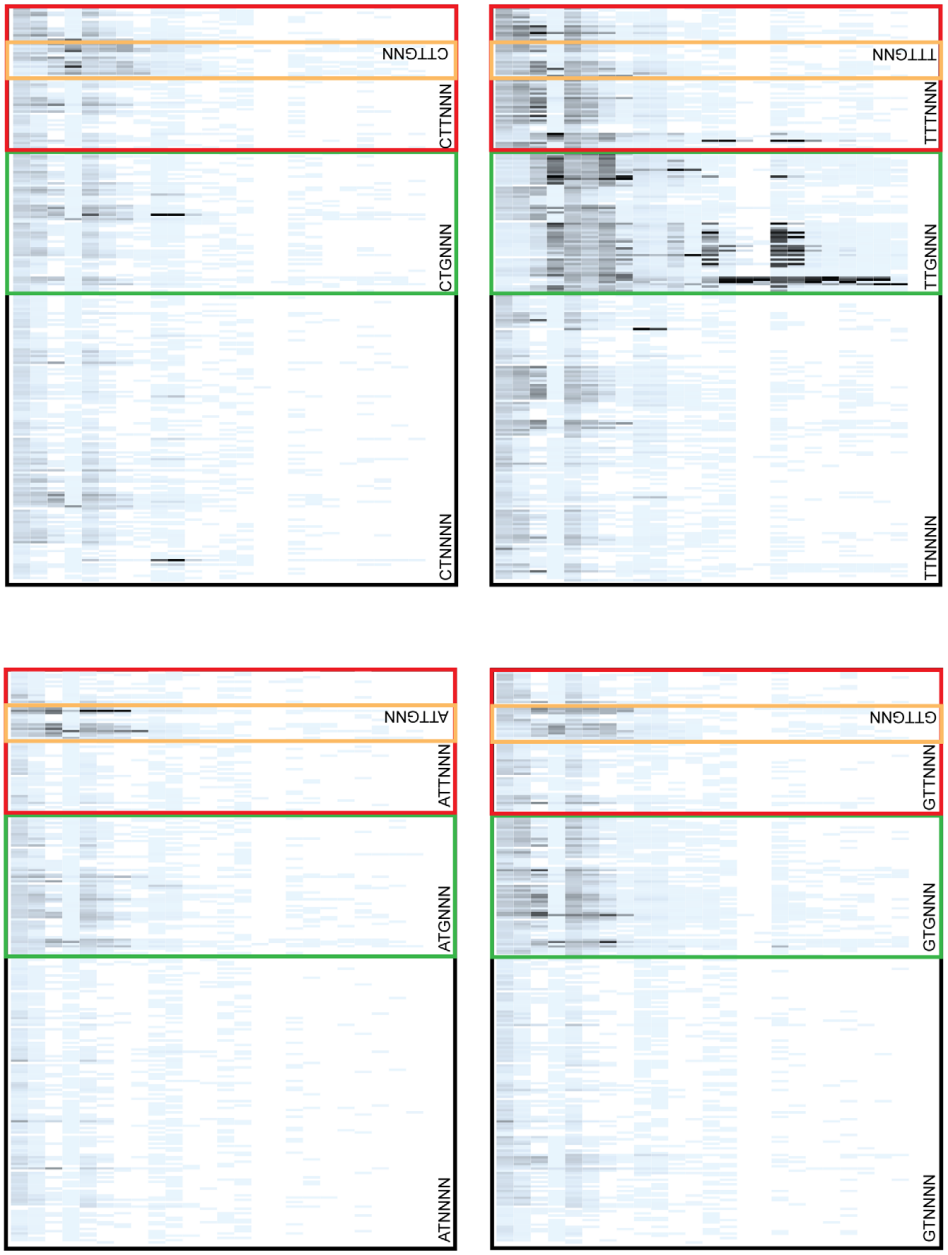
Expanded regions of functional sequence clusters. The colored boxes are expanded plots of the regions under the colored boxes in Figure S2. The sequence in the lower left corner of each box gives the common sequence to the sites in that box. The green, red and orange boxes are all contained within the black boxes. The average fitness of the sequence in the orange boxes are reported in Table 1. The selection conditions (y-axis) and the relative fitness scale is the same as in Figure 4.

Several competitions contained scattered low affinity sites with significantly higher fitness than the sites around them. We ordered all hexamers alphabetically (AAAAAA, AAAAAC, AAAAAG … TTTTTT) to see if there were sets of binding sites close in sequence space that had a high relative fitness, but not a high predicted affinity (Figure S2). We identified clusters of hexamers that contained a strong 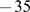 shifted one base to right (orange boxes in Figure S2 and Figure 5). That is, the second base of the randomized hexamer was the first base of the 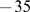 binding site. Differences in spacing between the 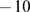 and 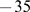 have been shown to affect the rate of initiation [5]. We tried to limit the number of 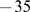 binding sites with sub-optimal spacings from our libraries by placing bases disfavored by the 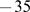 model at the positions flanking the randomized hexamer [11] (see Materials and Methods). Since the last two bases of the hexamer are fairly non-specific, it is difficult to exclude viable 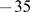
s with shorter spacings.

The fitnesses of the 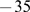 binding sites were reduced at shorter spacings compared to the larger optimal spacing, and only the strongest 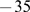 sites were viable and only under the mildest selection conditions (Figure 5). To quantify this, we calculated the average relative fitness of four sets of hexamers that had shifted 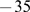 binding sites (Table 1). These sets of binding sites contained the 16 sites that had the consensus ‘TTG’ at the first three positions (positions 2–4 of the randomized hexamer) and a ‘G’ at the sixth position ( **TTGNNG**). This ‘G’ is the base immediately 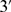 of the randomized 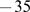 region, and is therefore fixed. The four sets only varied in which base was 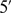 of the 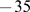, and should be the highest affinity sites at this spacing according to the 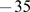 binding site model [11]. The average relative fitness was calculated across all experiments for these sequences (Table 1). The four sets had a similar average fitness to each other and a significantly higher fitness relative to 100,000 randomly chosen 16 hexamers (p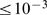), but on average were half as fit as the same set of sites at the optimal spacing ( **TTGNNG** ) and one third as fit as the 16 binding sites closest to the consensus ( **TTGANN** ) (**Table 1**).

**Table 1:** A shorter spacing between the −35 and −10 reduces fitness.

Sequence	Ave Fit
Random 16	0.009
A A**TTGNN G**C	0.047
A C**TTGNN G**C	0.042
A G**TTGNN G**C	0.041
A T**TTGNN G**C	0.045
A **TTGNNG** GC	0.091
A **TTGANN** GC	0.148

The average fitness was calculated for different related sets of hexamers. The ‘A’ at the first position in the sequence column is the base immediately 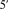 of the randomized 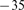 region (Figure 1). The sets of hexamers are the six bases (positions 2–7) flanked by spaces, and correspond to the randomized region. ‘N’ denotes a position that is varied in a set. The ‘GC’ at positions 8–9 are the two bases immediately 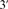 of the randomized region. The first four sets of hexamers (marked with orange boxes in Figure 5) contain a 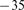 binding site that is shifted one base to the right relative to the optimal spacing (last two sets). The 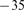 is bolded to show its position for each set. ‘Random 16’ is the average fitness for 100,000 randomly chosen sets of 16 hexamers.

**Figure 6:**
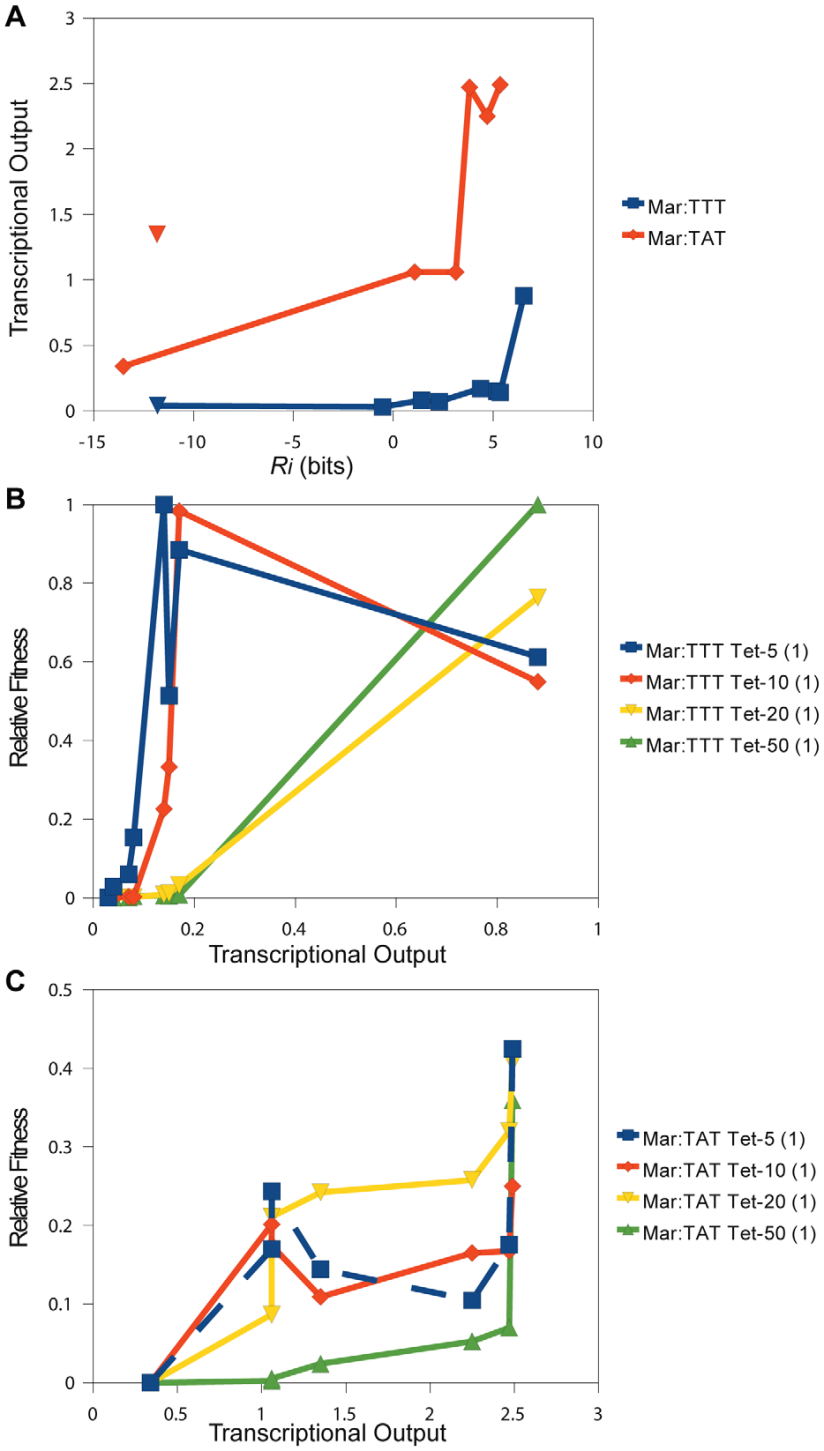
Direct comparison of relative fitness to transcriptional output. (A) The relationship between the predicted affinity (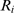) and measured transcriptional output is shown for different 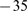 binding sites. This plot corresponds to the data in Table 2. The blue line is for 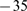 variants in the Mar:TTT context. The red line is for variants in the Mar:TAT context. The blue and red triangles designate hexamers that have a shifted 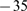. The relationship between transcriptional output (x-axis) and relative fitness (y-axis) for different 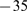 binding sites is shown for the (B) Mar:TTT and (C) Mar:TAT contexts.

To directly compare sequence activity to 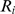 and relative fitness, we measured the transcriptional output of 8 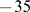 binding sites in the Mar:TTT promoter context and 7 in the Mar:TAT context by quantitative PCR (Figure 6). The sequences of these sites, their predicted affinities and their transcriptional activities are reported in Table 2. For both libraries, output generally increased with 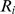. The data was best fit by a single exponential curve, but weakly; 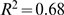 and 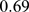 for Mar:TTT and Mar:TAT respectively (these values were only calculated for sites with an 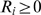 bits) (Figure 6A). Sites similar in sequence produced almost equivalent outputs. In the Mar:TTT context, *TTGC*GT, *TTGC*AG and *TTGC*TT vary only at their last two bases, and have similar activities (Table 2). In the Mar:TAT context, *TGG*AGC and *TGG*CTA vary at the last three bases and have the same output, and *TTG*CTC, *TTG*ATG and *TTG*CTT have similar outputs. We suspect the 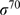 model is slightly overestimating the contributions of the last 3 bases of the hexamer, and this can account for inconsistencies between our predicted affinity and transcriptional output.

**Table 2:** Direct measurement of transcriptional output for different −35 binding sites by QPCR.

Sequence	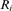	[*tet*]
CTTGAC…TTT	−11.8	0.04
AGTTAA…TTT	−0.54	0.03
TAGACG…TTT	1.41	0.08
TTGTGC…TTT	2.29	0.07
TTGCGT…TTT	4.36	0.17
TTGCAG…TTT	5.18	0.15
TTGCTT…TTT	5.34	0.14
TTGACT…TTT	6.53	0.88
CCGTTC…TAT	−13.51	0.34
CTTGCC…TAT	−11.82	1.35
TGGAGC…TAT	1.07	1.06
TGGCTA…TAT	3.12	1.06
TTGCTC…TAT	3.81	2.47
TTGATG…TAT	4.69	2.25
TTGCTT…TAT	5.34	2.49

The transcriptional output of different 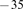 binding sites in the Mar:TTT and Mar:TAT contexts were determined by quantitative PCR. ‘Sequence’ is the sequence of the 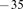 and the 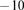 (TTT or TAT) in the expression construct. The Mar binding site was used in all constructs. ‘
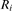
’ is the predicted binding strength for the 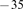 hexamer. ‘[*tet*]’ is the relative expression of the *tet* gene (see Materials and Methods).

Expression from the Mar:TAT context was much greater than from the Mar:TTT context. The weak TAGACG 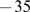 in conjunction with the consensus TATAAT 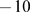 produced an output greater than the strongest 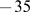 that we assayed in the Mar:TTT context, TTGACT. Additionally, the activity of the same 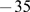 sequence (TTGCTT) in both contexts was 2.8 fold greater with the stronger 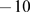. As seen in Figure 2 and Figure 3, these results indicate that differences in the 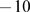 have a significant effect on transcriptional activity.

Two of the 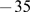 binding sites in the Mar:TTT context had an 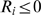 bits, and both produced the same weak expression level (Table 2). We expect all non-specifically bound 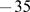
s to have this same output. One of these sites (CTTGAC) contained a strong 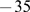 that was shifted one base closer to the 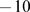, but showed no activity (blue triangle in Figure 6). Additionally, we characterized two 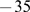 hexamers in the Mar:TAT context with an 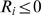 bits. One of these sequences (CCGTTC) showed a significantly reduced output relative to all other Mar:TAT sequences, but a high output relative to the Mar:TTT sequences. We expect this to be the transcriptional output for all non-specific 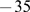
s in this context. The other sequence (CTTGCC) contained a strong 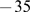 that was shifted one base to the right (orange triangle in Figure 6), but unlike the shifted site in the Mar:TTT context displayed high activity. This suggests that 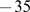
s with shorter spacings are only functional with the stronger 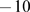, as seen in Figure 5.

There was a strong correspondence between transcriptional output and relative fitness for the 8 characterized 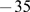
s in the Mar:TTT context (Figure 6B). At 5 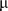
g/ml of tetracycline, fitness increased as a function of output for the 5 lowest expressing 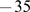
s and then slightly decreased for the 3 highest expressing. At 10 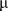
g/ml, the increase in fitness extended to all but the strongest 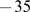, and at 20 and 50 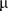
g/ml, fitness increased with output for all sequences. The cellular advantage for producing more of the tetracycline resistance protein may be outweighed by the cellular cost in low concentrations of drug [25]. This may explain this decrease in the overall fitness at greater outputs. The relationship between output and fitness for the Mar:TAT characterized 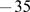
s was less striking (Figure 6C). At 5 and 10 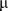
g/ml of tetracycline, we observed an initial increase in fitness from the lowest to the second lowest expressing 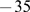, and then no consistent trend. It is important to note that the differences in fitness between variants in this context are relatively small, especially compared to the Mar:TTT examples, and there could possibly be no effect on fitness at these high expression levels in these low concentrations of drug. More data points are needed to determine this. At 20 and 50 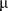
g/ml of tetracycline, we observed a general increase in fitness with output. Unlike in the Mar:TTT context, there was a gradual increase in fitness across these sites.

**Figure 7:**
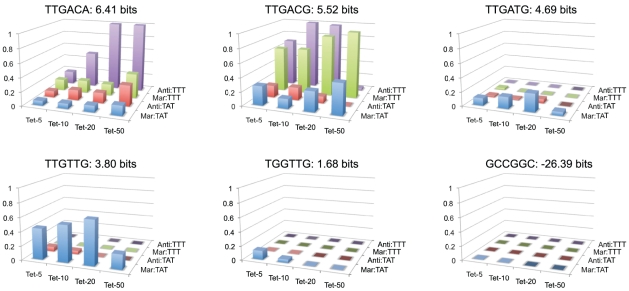
Fitness landscapes of individual −35 binding sites. The relative fitness of an individual binding site (z-axis) in different tetracycline concentrations (x-axis) and promoter contexts (y-axis) is shown. The name of each 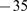 binding site and its predicted affinity are given above their respective landscape.

Fitness landscapes for individual hexamers across 16 different conditions are shown in Figure 7. We chose a series of five hexamers that decrease in predicted binding affinity from the consensus TTGACA, and differ from their neighboring sequence by a single nucleotide mutation. We also show a fitness landscape for the anti-consensus 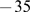 binding site GCCGGC. As expected the anti-consensus is not viable under any condition. There is an interesting contrast in the fitness landscape of the consensus sequence (TTGACA) to the weaker site TTGTTG. The consensus sequence shows a general increase in fitness to more stringent selective conditions, with a relatively low fitness in weak selective conditions. Conversely, TTGTTG is most fit in the weakest conditions and not viable at stringent conditions. TTGACG like TTGACA shows low fitness in the TATAAT 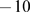 libraries, but has a greater fitness for most of the selections with the weaker TTTAAT 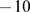 binding site, except for the most stringent. The fitness profile for TTGATG is weaker than expected for a site of that strength suggesting that its actual affinity may be lower than predicted. Regardless of our prediction of site strength, the difference between the TTGACG and TTGATG landscapes is large, illustrating how a single nucleotide mutation can radically change the fitness landscape of a 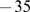 binding site.

**Figure 8:**
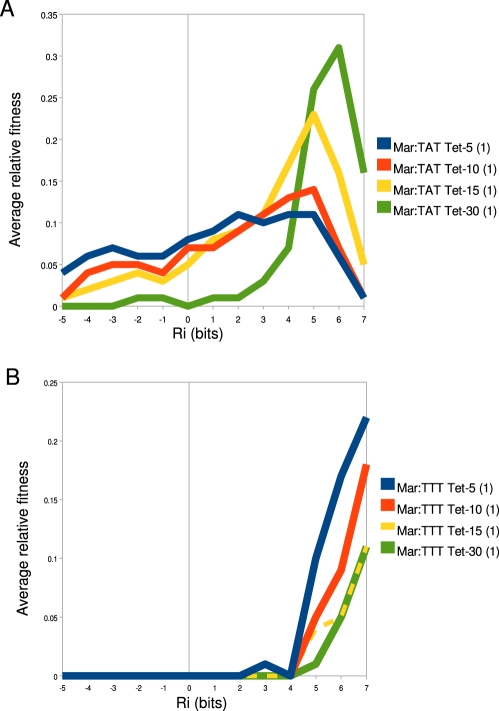
−35 fitness varies as a function of binding affinity. The average relative fitness for all 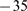 binding sites within a 1 bit range of affinities is shown. The value at 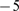 is the average relative fitness for all sites 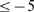 bits. 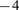 is the average binding fitness for all sites 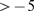 bits and 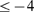 bits and so on for all 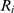 ranges. The key to the right of each graph identifies the library that corresponds to a given line in that graph. Differences in 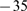 fitness as a function of increased tetracycline for (A) Mar:TAT libraries and (B) Mar:TTT libraries are shown.

To better understand how binding site strength correlates with relative fitness in different promoter and environmental contexts, we calculated the average relative fitness for all sites within 1 bit bins (Figure 8). For the Mar:TAT library (Figure 8A), we observed that the 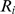 range that has the greatest average fitness is not the highest one. We did observe an increase in the strength of the optimal fitness range as we increased the selection concentration of tetracycline, but for all tetracycline concentrations we saw a decrease in fitness at the highest range of binding sites. For the Mar:TTT library, we observed a general increase in relative fitness as a function of binding site strength for all tetracycline concentrations. Interestingly we did not observe the decrease here as we observed in Figure 6B. We did observe a similar decrease in fitness at higher information sites for the Anti:TAT library at 5 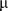
g/ml tetracycline, but not at higher concentrations. The Anti:TTT library only showed an increase in fitness at higher binding site strengths (data not shown).

## Discussion

To decipher *cis*-regulatory information and subsequently understand how it evolves, we need to be able to experimentally associate expression phenotype to genotype for large libraries of sequences. While there has been some success in doing this [12], these datasets are still extremely challenging to generate because it is difficult to maintain genotypic information in bulk reactions, requiring a large number of independent assays. Here we were able to overcome this problem by measuring the abundance of a genotype in a competed population of promoters, where cellular fitness is a function of its transcriptional phenotype (production of the *tet* gene). Given a mapping of phenotype to genotype for large libraries of sequences, it is still difficult to parse out the effects of single nucleotide differences on transcription since the rate of initiation is dependent upon many variables. Here we reduced this problem by generating libraries of promoters that only differ by the sequence of a single binding site (the 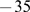). The method worked well. For the first time, we were able to generate experimentally determined fitness landscapes for a large set of sequences in multiple promoter and environmental contexts. These data give insight into both the mechanism and evolution of transcriptional regulation at the level of an individual binding site.

### Promoter fitness varies as a function of 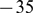 binding site strength

The fitness of the transcriptional output of a binding site is a complex function of the cellular gain and cost associated with the production of expressed gene [25]. The cellular gain in our synthetic system is the increased ability to export tetracycline from the cell. The cellular cost is the toxic effect of over-expressing the tetracycline efflux pump [26], [27]. While we do not fully understand the absolute relationship between binding site strength, transcriptional output and the fitness of that output, clearly these things are related (Figure 8, Figure 6) and highly context dependent (Figure 7).

The relative frequency of recovery of a 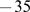 binding site in a competed population is dependent upon two variables, 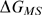 (Minimum Viable Stability) and 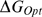 (Optimal Stability). 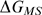 is the minimum stability of the initiation complex needed to produce enough of the *tet* gene to survive. 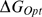 is the stability of the initiation complex that produces the maximally fit output given a concentration of tetracycline. For a 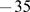 to be viable in our selection, it must have an affinity that in combination with the other binding sites produces an initiation complex stability that is stronger than 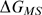. As the strength of the other sites or the output requirement changes, so does the boundary of the minimum viable 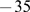 binding site strength. This is indeed what we observe in Figure 4 and Figure S1. As we increased the concentration of tetracycline (decrease 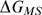) or as we decreased the strength of the 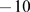 or MarA binding sites, only stronger 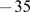
s remained in the selected population. This is also illustrated in Figure 2 and Figure 3 as a decrease in the variability of the population and a convergence on the consensus sequence at more stringent (energetically demanding) selection conditions. Compensation in binding energies between sites to produce similar stabilities has been previously predicted computationally for 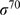 binding sites [11] and is shown clearly here. Interestingly, the information content of the competed populations increases linearly as a function of tetracycline concentration over the range of 5 to 30 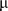
g/ml and levels off at 50 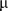
g/ml for both the Mar:TTT and Mar:TAT libraries (Figure 3). We are not sure why the information content levels off. One possibility is that we are approaching the maximum stability where the transcriptional initiation rate is limited by the stability of the closed complex.

The most fit 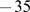 in a given context should have an affinity, that in combination with the other binding sites, equals 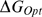. We expect that fitness will increase with the overall stability of the initiation complex from 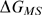 to 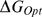. We observe this qualitatively for libraries containing the weaker TTTAAT 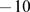 binding site or libraries selected at high concentration of tetracycline. Here, sites generally increase in fitness as a function of binding site strength (Figure 4, Figure S1). Some 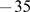 sequences show an unexpected high or low fitness compared to their neighboring sequences with similar predicted affinities. These could be partially explained by insufficient sequencing depth, but we expect to a small degree since technical replicates suggest that for most conditions our depth gives an accurate representation of the population. Another possibility could be that some promoters may be under or over-represented in the initial library. We expect that to some extent these discrepancies are due to inaccuracies in the binding model that we used. A comparison between 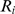 and transcriptional output suggests that the model may be slightly overestimating the energetic contributions of the last three bases of the hexamer to binding site strength (Figure 6). A large number of sequence anomalies can also be attributed to 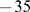 binding sites with shifted spacings relative to the 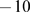 (Figure 5).

When the average fitness is calculated for binding sites with similar affinities (reducing the effects of anomalous 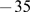
s), we see a smooth relationship between fitness and binding site strength (Figure 8). In strong selection conditions (high tetracycline concentration, weak 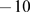), 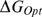 exceeds the maximum stability that can be accessed by only varying the 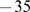 binding site, so here an increase in 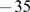 binding affinity always increases fitness (Figure 6B and 6C, Figure 8B). In weak selection conditions (low tetracycline, strong 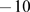), the optimal 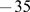 binding site does not appear to be the strongest (Figure 6B, Figure 8A). That is, 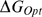 is within the range of affinities that can be accessed by changing the 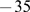. The additional energy from the 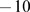 presumably shifts the distribution of outputs for the 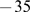 binding sites into a range where there is no longer an increased advantage or even a disadvantage for transcribing that much *tet*.

Overall, we observed a large and continuous range of fitnesses suggesting a similar scope of potential outputs can be evolved or engineered by solely mutating the 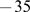. Fitness landscapes of individual 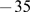 sequences illustrate the large effect on fitness by even a single mutation (Figure 7). It is not clear what the maximum stability of the initiation complex is where increases in stability will no longer increase output (closed-complex stability is not limiting). It has been shown for some promoters that too strong of an interaction can actually decrease transcriptional output, presumably because it is difficult for the polymerase to dissociate from the DNA [28]. A decrease in fitness from the highest affinity consensus binding site compared to a single base pair mutation of the consensus in the Anti:TTT context (Figure 7), suggests that the range of affinities of 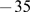 binding sites alone does not exceed that maximum. There may have been selection on 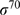 to keep the range of 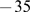 affinities below this maximum, to maximize its output range.

### Binding sites do not contribute equally to the fitness of the promoter

The relative contributions of the 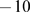 and MarA binding sites do not appear to be equivalent. A single mutation in the second position of the consensus 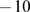 greatly reduces the variability of the 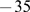 binding site populations. Whereas completely removing the MarA binding site has a significantly reduced effect. This suggests that binding at the 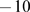 contributes more to the stability of the initiation complex than does binding by MarA. The decrease in effect from the MarA site could be related to the energetics in the contact with the 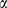
CTD which we do not understand [11], or MarA expression could be low resulting in a low occupancy of the site.

The significant effect of mutating the 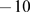 on transcript production is clearly shown in Figure 6A. The expression levels of all 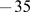
s in the Mar:TAT context, except for the non-specifically bound one, are greater than the expression from the most active 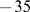 in the Mar:TTT context that we characterized. This suggests that differences in the 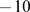 may contribute more than differences in the 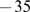 to the overall output. Open complex formation occurs through melting at the 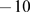 [11], [29], [30]. A mutation in the 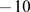 sequence could have a greater effect on the rate of initiation because it could lead to both a change in promoter stability and the rate of open complex formation. We expect that regardless of whether differences in the 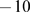 affect the stability of the closed complex or open complex formation, selection on the 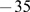 will be on its binding site strength. The larger range of outputs in the Mar:TAT context compared to the Mar:TTT context suggests some cooperativity between sites (Figure 6A). We do not have enough data to determine to what extent.

As previously mentioned, the spacing between the 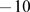 and 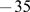 can affect the rate of initiation [5]. While we tried to minimize the number of 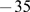 binding sites with alternative spacings from our library, this proved difficult because the last two positions of the hexamer are fairly non-specific. We observed that 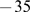 binding sites were viable with a 1 bp shorter spacing relative to the 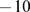, but only in weak selective conditions (low tetracycline, strong 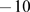 and MarA binding sites) and only the strongest sites (Figure 5). This was confirmed by quantitative PCR, where we observed that only in the Mar:TAT context, could shifted sites produce an output above that of a non-specifically bound 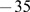 (Figure 6). The additional energy of the 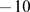 may be able to compensate for the energetic cost of binding the 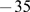 with a sub-optimal spacing [11]. We observed a similar average fitness for related sets of binding sites with a shifted 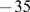 (Table 1), suggesting that differences in the position 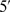 of the 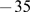 do not affect transcriptional initiation. These sets of binding sites were on average about half as fit as the same set of sites with the larger optimal spacing, suggesting that differences in spacing significantly decrease transcriptional activity.

## Materials and Methods

### Binding site library construction

We placed the tetracycline resistance gene (*tet*) under control of a MarA-activated 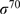 promoter on the *E. coli* plasmid pBR322. pBR322 has several advantages: (1) It confers resistance to both ampicillin and tetracycline, allowing for maintenance of the plasmid to be either independent of or dependent on the promoter of *tet*. (2) It is a relatively low copy plasmid (15–20 copies per cell) [31]. This eliminates the high expression of *tet* associated with large copy numbers. We generated four promoter libraries where the 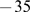 was randomized and contained either one of two MarA and 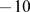 binding sites (Figure 1).

Variability in the relative spacing between binding sites can affect the rate of transcription [5], [6]. We designed the promoter insert to strongly favor a single spacing between the 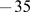 and the 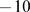 to avoid having to consider spacing effects on the fitness of the promoter in the analyses. We used the optimal spacing between the 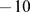 and 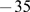 [11], where deviations from this spacing would result in a decrease in binding affinity. Additionally, the two bases immediately 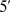 (‘CA’) and the two bases immediately 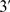 (‘GC’) of the 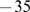 hexamer are disfavored at the first and last two positions of the 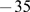 respectively [11], further reducing the possibility of strong 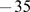 binding sites with different relative spacers. The sequence between the 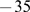 and MarA binding site is a slight variant of the sequence found between the MarA site and the 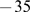 in the *mar* promoter [22]. We shortened the spacer by one base at the 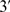 end to have the disfavored ‘CA’ immediately adjacent to the 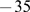. Martin *et al.* showed that this shortened spacing has a minimal effect on the degree of MarA activation [6]. We also changed three bases in the spacer to create a *Bst*BI site (TTC**ATT** is now TTC **GAA**).

The weaker 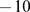 (TTTAAT) in the promoter of the *tet* gene was mutated to the consensus 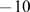 (TATAAT) by QuickChange according to Zheng *et al.* [32]. These two pBR322 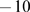 variants, pBR322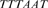 and pBR322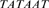, were used for subsequent library construction. The 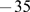 of the *tet* gene on pBR322 is flanked by two unique restriction sites, *Eco*RI and *Cla*I. These sites were used to clone in MarA binding site and 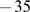 variants as described below.

The randomized 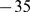 library inserts were created by DNA synthesis (Integrated DNA Technologies). Variation of the 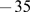 binding site was done by mixing equal quantities of each base at those positions. Two library inserts were synthesized that contained either the stronger *mar* MarA binding site [22], or the non-specific anti-consensus MarA binding site. The latter has the least frequently observed base at each position based on the MarA binding model (model not published but generated from sequences in [6]) and should not be bound. These inserts will be referred to as Ins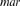 and Ins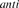. The DNA was made double stranded by second strand synthesis with Klenow (NEB), and the fragments were purified with a QIAquick PCR purification kit (Qiagen).

pBR322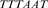, pBR322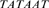, Ins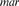, and Ins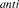 were cut with *Eco*RI and *Cla*I (New England Biolabs) for two hours at 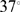
C and gel purified using a QIAquick gel extraction kit (Qiagen). All four combinations of plasmids and inserts were mixed and ligated overnight at 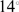
C with T4 DNA ligase (NEB) generating 4 libraries (Mar:TAT, Anti:TAT, Mar:TTT and Anti:TTT). The ligated libraries were transformed by electroporation into DH10B cells (Gibco BRL), and plated on 100 ml LB+30 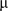
g/ml ampicillin plates. The number of transformants for each library was *ca.* 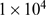. The colonies were suspended from the plate in 10 ml LB, and mini-prepped using a QIAquick miniprep kit (Qiagen).

### Promoter competition

Libraries were transformed by electroporation into the *E. coli* strain DH10B (Gibco BRL). The number of transformants was *ca.* 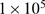 as determined by plating. After transformation, cells were recovered in 500 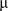
l LB for 1 hour, and grown further in 5 ml LB+30 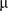
g/ml of ampicillin overnight at 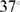
C, with shaking at 225 RPM. Fresh 5 ml LB cultures containing from 5 to 50 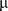
g/ml of tetracycline were inoculated with 100 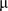
l of the promoter libraries grown overnight. Promoter libraries were competed against each other for 24 hours at 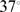
C, with shaking at 225 RPM. Plasmids were purified from the competed libraries using a QIAquick miniprep kit.

### Measurement of transcriptional output by quantitative PCR

The Mar:TTT and Mar:TAT libraries were plated on LB agar plates containing 0 to 100 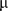
g/ml of tetracycline. Individual colonies were sequenced from these plates, and 8 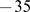 variants in the Mar:TTT context and 7 variants in the Mar:TAT context were chosen that covered a large range of predicted binding strengths for further analysis. 5 ml LB cultures containing 30 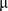
g/ml of ampicillin were inoculated with *E. coli* containing a single 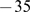 binding site variant and grown overnight. A fresh 5 ml LB+30 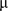
g/ml ampicillin culture was started at 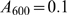 and grown to an 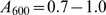. 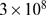 cells were added to RNAprotect Bacteria reagent (Qiagen), and RNA was purified using the RNeasy Mini kit with on-column DNase digestion (Qiagen). cDNA was made from 2 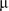
g of RNA using the Superscript III RT kit (Invitrogen). QPCR was performed with the SYBR green mix from NEB. QPCR primers specific to the *tet* and *gyrA* gene were both used. The relative expression of the *tet* gene was determined by the ratio of *tet* transcript abundance over *gyrA* transcript abundance for each sample. A serial dilution of the Mar:TTT, TTGACT 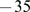 sample was used as a standard for both primer sets. The expression of the *tet* gene for all variants was calculated relative to this. All sequences used, their predicted affinity (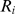) and the expression values are reported in Table S2.

### Solexa sample prep and sequencing

Conversion of pBR322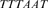 to pBR322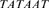 destroyed a *Hind*III site that overlapped the first two bases of the 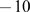 hexamer. Libraries that contained the wild type 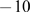 (TTTAAT) were digested with *Hind*III and *Pvu*I (NEB) for 2 hours at 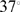
C. pBR322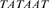 libraries were digested with *Cla*I and *Pvu*I (NEB) for 2 hours at 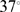
C. 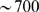 base pair fragments were gel purified for all four libraries using the QIAquick gel extraction kit. Excised fragments from all four promoter libraries, selected at a single tetracycline concentration, were mixed at equal concentration. Solexa libraries were then generated from this mixed population.

The Illumina genomic library protocol was slightly modified (Illumina, Inc.). We used a 1∶10 dilution of the Solexa genomic adapter, and ran the PCR for 16 rounds. We gel purified the final product after the PCR step instead of before as suggested. This allowed the removal of potential adapter contaminants. Sample purity and concentration were measured using a Bioanalyzer (Agilent Technologies). A 45 bp single-end run was performed on a GAII machine according to the Illumina protocol.

### Analysis of fitness data

For each tetracycline concentration, the reads were identified as originating from one of the four promoter types. We used only those sequences that had an exact match to 14 or 21 specific bases that flanked the −35 region for the TAT and TTT libraries respectively. We did this to ensure that this sequence was not mutated, the spacing between the −10 and −35 was not changed, and to increase our confidence in the accuracy of the −35 sequence. We used 7 additional bases for the TTT libraries because those libraries were cut 7 bases further from the −35 than the TAT libraries. These additional bases were used to determine which 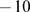 variant was present for that sequence. Additionally, we required an additional 10 bases before and overlapping the MarA binding site to exactly match to confidently distinguish between the Mar and Anti libraries. The number of reads for each competition that pass these criteria are reported in Table S1.

Each 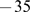 was counted for each competed library at a tetracycline concentration. To determine the relative fitness of a 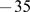 in a competed population, the number of reads containing that 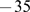 was divided by the number of reads of the most frequently observed 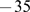. For two of the competitions, Anti:TAT Tet-5 and Anti:TAT Tet-10, three hexamers (TGCCCA, TCCATT and CTGGAT) were disproportionally high relative to the others. Interestingly, if two of these hexamers are put in the context of the promoter sequence, CA-TCCATT-G is only one base different from the reverse complement of CA-CTGGAT-G (C-ATCCAG-TG). The hexamer sequence is separated from surrounding sequence by ‘-’. These sequences may encode for the binding site of some unknown factor which may explain their increased fitness. At greater tetracycline concentrations though, these were observed much less frequently. For these competitions, the fitness of the hexamers were calculated relative to the fourth most frequently observed hexamer.

Sequence logos were generated from the alignment of all 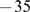 reads for a single library at a single tetracycline concentration using the **delila** software [33].

### Inference of binding affinity

We used the program **scan** to predict the relative affinity (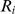) of 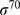 to each 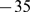 hexamer. Briefly, **scan** compares an individual sequence to an information theory based 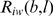 weight matrix and sums the information contribution of each base across all positions in a site [2]. The 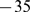 weight matrix that we used is the one generated from 401 experimentally verified 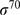 promoters in *E. coli* presented in [11] and is given in the supplemental materials of this paper (Table S3).

There are several advantages to this approach. First, the weight matrix is generated from a large number of experimentally verified promoters, and should not be skewed by binding site selection biases [34]. Second, 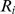 has been shown experimentally to be directly proportional to 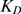 and more specifically 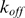 [4]. Third, the information theory approach predicts a clear demarcation between specifically and non-specifically bound sites at 0 bits [24].

## Supporting Information

Figure S1 Expanded region of Figure 4 with *Ri*≥0 bits. The relative fitness scale is the same as in Figure 4.
(2.07 MB EPS)

Figure S2 There are distinct clusters of functional sites in sequence space. This is a similar plot to Figure 4 except the −35s are ranked alphabetically. The first hexamer (far left on the x-axis) is AAAAAA, then AAAAAC, AAAAAG, AAAAAT, AAAATA, *et cetera*. The colored boxes correspond to the zoomed in regions in Figure 5. The relative fitness scale is the same as in Figure 4.
(5.70 MB EPS)

Table S1 Number of sequenced promoters for each competition. The number of Solexa sequenced promoters is given for each library at each concentration of tetracycline.
(<0.01 MB XLS)

Table S2 Number of Counts and Relative Fitness of each −35 for each conditions, sorted by fitness.
(4.02 MB XLS)

Table S3 −35 weight matrix used for analysis. The −35 weight matrix was built from 401 experimentally verified σ^70^ binding sites and originally presented in [11]. ‘Count Matrix’ gives the count of each base at each position (l) in the −35 alignment. ‘Information Matrix’, gives the individual information for each base at each position [2].
(<0.01 MB XLS)
